# On the Influences of Non-Specific Emanations on the Public Health; Are They Deleterious?

**Published:** 1871-04

**Authors:** Wm. C. Roberts

**Affiliations:** Vice-President New York Academy of Medicine


### On the Influences of Non-Specific Emanations on the Public Health: Are they Deleterious?

By Wm. C. Roberts, M. D., Vice-President New York Academy of Medicine.

Cheerfully admitting the great noisomness of these public nuisances, I design, in this paper, to consider, as candidly as I can, and as derived from recognized authorities, their pestilential influences in a scientific point of view.

In a paper read before the Academy in a former year, I endeavored to show that noisome smells, or effluvia, or fetid emanations, from various sources, both of animal and vegetable decomposition, or the gases which produce these smells, or in which they resided, were not necessarily, and in all cases, injurious to the health of individuals, nor communities ; and fortunate it is that it is so ; that, except under certain circumstances, they did not engender disease, nor were pestilential, nor detrimental to health, and that their importance in this respect had been overrated ; and that, when non-specific, however offensive, they were for the most part innocuous.

In certain idiosyncrasies, when intense, they do occasionally induce nausea, vomiting, diarrhoea, cholera-morbus, dysentery even, and fever of a typhoid type, acting partly through the brain and nervous system, partly through the blood.

In some recorded instances, several persons residing in the same limited space, and exposed equally to these emanations—say in a school, or an asylum—had been similarly attacked, and deaths even had occurred among them ; but the diseases so produced were neither specific nor contagious, and were limited in extent to the locality. I endeavored to show that in the filthiest and most feted streets in this metropolis, Orange, Baxter, Mott, Mulberry, and Elizabeth, and others, where the air reeked with the tainted odors of slaughter-houses, etc., the inhabitants did not suffer in a greater degree than those of others, and adduced other instances of the comparative salubrity of persons engaged in offensive manufactures.

No one can doubt that a gas, or a concentration of gases, incapable of supporting life, must be deleterious and deadly to persons inhaling them, without inhaling at the same time a sufficient amount of oxygen to counteract their noxious influence; that they produce syncope, or sudden or slow asphyxia; but they are rendered innocuous by their speedy and general diffusion in the air, and thus, offensive as they are, are greatly shorn of their morbific influences. Such an atmosphere, however, is not one that would be selected either for health or pleasure, and the propriety of their removal cannot be questioned.

Not long ago, while small-pox was prevailing very extensively in the city, on both sides, I had occasion to pass down West Thirty-second Street, between Seventh and Eighth Avenues, which was encumbered with masses of festering filth, and stunk as offensively I think, as any streets I have ever passed through; yet the case-book of the Board of Health did not show that any special susceptibility to the prevailing endemic existed in this locality, nor in many other streets as filthy, as compared with wide and comparatively cleanly thoroughfares. To the instances of the correctness of this view, which I have already cited (see *Bulletin of Academy*,) I desire to add some others with which my reading has furnished me, whereby the true influences of noxious emanations may be rightly appreciated.

In the *British Medical Journal*, vol. ii., p. 356, 1864, the reader will find a brief report of the annual meeting of the British Association for the Advancement of Science, at Bath, Eng., September, 1864. On this occasion Dr. J. Hughes Bennett laid down for debate and discussion the following propositions:

1. Atmospheric air strongly impregnated with odors of different kinds was *not necessarily injurious to health.* No injury to health had been shown to result from an establishment in Paris for the distribution of manure; and the condition of the river Thames had not been productive of the slightest effect on the health of London. Naples is a very volcanic neighborhood, and the drains throw off large quantities of sulphurated hydrogen, which emit a most offensive odor. Yet Naples is not more subject to typhoid fever than any other city. The hospitals were stinking and filthy, yet no fever was caused in that way. The most pestiferous fevers prevail endemically in places where there are no bad smells, and are not attributable to such causes. Carburetted hydrogen, which has no smell, is as deleterious as sulphuretted hydrogen, which smells very badly. The smell of the water of Leith will "knock down the deil;" but it is not pretended that any person had suffered inconvenience from the bad smell, and its banks were the healthiest parts of the city. "Smells," said Dr. B., "as smells, were neither injurious to health nor were they a nuisance to those who lived among them. They became accustomed to them, and may even learn to like them, though this is not universal." Dr. B. goes on to say that the deleterious gases arising from effluvia were only injurious by being carried into the blood, and to this end they must be sufficiently concentrated, and the atmospheric air proportionately diminished. The fish in all rivers are not destroyed by sewage. Some, as birds on carrion, and pigs and eels on garbage, grow fat on it. Typhoid fever cannot be proved to originate from the fermentation of sewerage water. There were not wanting some coincidences respecting drains; but there were innumerable cases of emanations that had never causeed epidemics, to counterbalance those on the other side. Epidemics have not been diminished by costly drainage, as is seen in Paris. In Edinburg, old town, there was, until lately, no drainage, and typhoid fever is unknown there. Dr. Livingstone, the distinguished African explorer, believed it was most important to know that stinks were not the causes of fever in Africa. He stopped his suite all night at a place down the Nyamzi, where the water, as it came out of a marsh, was as black as ink, and had a most abominable smell, turning the paint on the ships white, etc. This phenomena did not produce illness in the crews, nor was it known to do so among the natives. It would, he said, be a great mistake to suppose that fevers came from the presence of bad smells. Dr. Kirk said that in similar localities in Africa there were never any serious fevers. Dr. McAdam gave a modified opinion, but said that bad smells were not necessarily injurious, and that chemists lived in the midst of sulphuretted hydrogen, and without experiencing any ill effects from it. But other injurious things, not isolated, might be evolved from putrefying matters after, or without sulphuretted hydrogen. He quite admitted that there were other causes than drainage affecting the mortality and general health of the people.

Dr. William Budd believed that Dr. Bennett was entirely right in laying it down as a fundamental principle that foul gases had no power to generate a fever; the sewers never generated the fevers, nor their poisons, but they distributed them. The specific poisons are eliminated from the bodies of the sick, and carried into the sewers. No doubt, therefore, the process of removal and destroyal of specific exuviæ, before they became cast off and set at large in the community, was proper, and tended to prevent the spread of diptheria, typhoid fever, and even scarlatina; and he proposed a resolution which might be advantageously laid before the Association on this occasion: "That it is desirable that a committee should be appointed to report to the Association, at some future meeting, whether the specific agent, which is the cause of typhoid fever, be ever generated *de novo* out of common sewage, or whether sewers only propagate this fever by the dissemination of the germ in liquid evacuations from persons affected with the disease:" which was carried; but whether such report has since been made, I cannot say.

Dr. Richardson said that Dr. Snow, some years ago, pointed out the difference between a bad smell and a poison. So far as this distinction went, he entirely concurred with Dr. Bennett. Dr. Bennett said that he had succeeded in the object he had had in view, which was to provoke discussion. He had never said that bad smells were good things. He disliked them as much as anybody; but the great point he wanted to force forward was, "that this effect of smell upon the health of the public had been greatly exaggerated;" which is entirely my own opinion.

Dr. Parkes ("Manual of Practical Hygiene") thinks that the water drunk is more injurious than the air breathed; he points out the brevity of incubation of typhoid fever, when conveyed by water, as compared with that conveyed by air; he by no means believes that cholera is alone conveyed by drinking-water. Yellow fever may, but no other zymotic disease can be conveyed in this way. He says, "We now know that, unless the specific cause be present, *no mere foulness of air will produce a specific disease;*" and adds, most truly, that accurate statistical inquiry, on a large scale, alone can prove what may be in reality a serious depreciation of general health. He shows that, in spite of very free ventilation, the poisons of small-pox and scarlatina will long preserve their power of re-producing the same disease. He believes that sewer air is productive of mischief, rather of the digestive tube than of the pulmonary system. Without absolutely denying the possibility of the *ne novo* origination of typhoid fever from simple sewage matter, he is convinced that sewers afford the channel of communication when containing the specific poison of the stools.

If we examine the papers of that very distinguished epidemiologist, Dr. William Budd, of Bristol, England, in the *British Medical Journal*, 1861, we shall find him a firm asserter of the innocuousness of mere smells, a decided contagionist, and an utter disbeliever in the *possibility of any generation of specific disease de novo*, in which he concurs with Watson and Graves. In the first place, he objects to the term "pythogenic" (typhoid) fever, as a disease born of putrescence, as depending on an untenable theory. It cannot be pure sewage that causes the fever; it must be *sewage of a particular kind.* In North Lawton, severely visited in 1839, there had not been in ten years more than one case of fever; and yet the hypothetical "fever-demon of the sanitarian" stunk as loudly during that long period of entire exemption, as he did when nearly one-sixth of the whole population was struck down with fever. "If he were not there in person, he surely had no business to smell so badly." This is true generally of places everywhere, and in villages particularly, because of the deficiency of or defect in sewerage; the evacuations of diseased persons are thrown out upon the soil; showing that these impurities have no power of themselves to cause fever, *unless when charged with specific poison.* These discharges in large cities are quickly swept out of harm's way; in country places they accumulate, day by day, upon the open soil, and envelop the household and the neighborhood. Hence the vital importance of *disinfecting* such exuvia, of which abundant proofs are furnished in the subsequent paper.

But, of all the striking instances known of the innocuousness of *mere smells,* however offensive, that which may well be called the greatest on modern record, quoted by Dr. Budd, must here be cited. The river Thames, which bisects the city of London, began, in the hot months of 1858 and 1859, to stink loudly. It emitted what Falstaff, after his experience in the buck-basket, called the "rankest compound of villanous smells that ever offended nostrils." It was an epidemic stink, *epi demos,* upon the people. It needed no particular susceptibility for its recognition; not the fastidious delicacy of Hotspur's fop, who complained that a "beggarly unhandsome corpse should be brought betwixt the wind and his nobility;" nor the keen scent of the amiable and philanthropic Florence Nightengale, who "saw with her eyes and smelt with her nose," the small-pox, in the course of formation, in the wards of a crowded hospital. It was as palpable to the coster-monger, as he walked beside his donkey, as to the Lord High-Chancellor, in robe of state, seated on the wool sack. For the first time in the history of man, the sewage of nearly two million people had been brought to seethe and ferment under a burning sun, in one vast *cloaca,* lying in their midst. Stench so foul had never before ascended to pollute this lower air. The committee-rooms of Parliament were rendered habitable only by the use of deodorizers; the law courts were broken up; the river steamers lost their traffic, and travelers went many miles around, to avoid crossing the bridges. "India is in revolt, and the Thames stinks," were the two great facts, coupled together by a distinguished foreign writer, to mark the climax of a national humiliation. Pestilence, cholera, and fever, were loudly predicted by persons of all classes; a case of malignant cholera did occur in the person of a Thames waterman, and was the key-note of a general alarm. But, did it occur? Were these dire anticipations, so naturally and confidently ascertained, realized? On the contrary, the health of the metropolis remained remarkably good, and fever, diarrhoea, and dysentery, which last two might certainly have been expected, diminished in comparison with the preceding year. Dr. McWilliams, a water-side supervisor, says: "The stench from the river and docks was in nowise productive of disease, however noisome; on the contrary, there was less of that form of disease to which foul emanations are supposed to give rise than usual."

Of two places in like sanitary condition, one may be the seat of virulent fever, and the other, perhaps the worse of the two, remain perfectly free. Exbourn, four miles from North Tawton, was the filthiest of places in the same season, and escaped; but afterward a low fever *was imported,* and spread virulently, when it had died out in Tawton. This is the history of small-pox, measles, scarlatina, and cholera; the same alternations of slumber and activity, of prevalence in certain places; the same successive invasion of neighboring places. The specific morbid cause, always existing, is transmissible from place to place, breeds as it goes (zymosis,) and then dies out, or becomes dormant, without leaving any sign to mark its track or existence, until again awakened into activity. Diseases so engendered are essentially contagious. "To conclude," says Dr. Budd, "on the evidence usually assigned for such a belief, that specific poisons, possessing the habitudes that belong to their history, are bred in every cesspool, or ditch, in which there is seething rottenness, or decomposition, is akin to the ancient belief that mushrooms are bred of cow-dung, alligators of the mud of the Nile, or bees, as Virgil sung, out of the entrails of a putrid ox; and signs are not wanting to show that the times are not far distant when the belief in question will take its place in that limbo of discarded fallacies to which these other superstitions have long been consigned."

Since, then, it seems very clear, from what has preceded, that mere stenches, from whatever cause proceeding, or however vile, offensive, and concentrated, are injurious only in a few limited instances, in persons of very susceptible organizations, in whom they produce certain forms of gastro-intestinal disturbance, and cannot be said in any general sense to be pestilential, injurious to health, or detrimental to life, it follows that all that is necessary in the case of gas-houses, fat melting, bone-burning establishments, slaughter-houses, and such other stench generators as are public nuisances, is, that they should be removed from the midst of populously inhabited neighborhoods, and *deodorized*, to render them generally perfectly innocuous. Sewers and cesspools, etc., because they *may* contain, besides the mere elements of decomposition and fetor, materials, derived from human excreta, holding in themselves the specific poison of disease, and susceptible of zymotic reproduction, and a contagious principle, which may diffuse emanations capable of propogating and extending pestilence over a very wide extent of surface, as many recorded examples show, should be not only deodorized, but *disinfected* as well.

All this, I am proud to say, has been done daily for years past, to a very great extent, as their annual reports will show, by the late liberal and enlightened Metropolitan Board of Health, under the supervision of its former energetic and scientific sanitary superintendent, and will be continued, I do not doubt, under his worthy successor in office.

In this way only can threatened pestilence be excluded, its spread when existent limited, its seeds destroyed, and its multiplication arrested. Such has been, to my certain knowledge, during the past year, the effect of measures devised in a spirit of true philanthropy and zeal for the public health and safety, and carried out energetically with the aid of all well recognized resources of sanitary science, sequestration, cleansing, and *disinfection*. — *New York Medical Journal.*

